# Persistence of left superior vena cava: a rare cause of hemodialysis tunneled catheter malposition

**DOI:** 10.1590/2175-8239-JBN-2020-0263

**Published:** 2021-05-28

**Authors:** Afonso Santos, Ana Gaspar, Anna Lima, Catarina Brás, Pedro Campos, Célia Madeira, Ana Nassauer Mónica, Karina Soto

**Affiliations:** 1 Hospital Prof. Doutor Fernando Fonseca, Departamento de Nefrologia, Amadora, Lisboa, Portugal.; 2 Hospital Prof. Doutor Fernando Fonseca, Departamento de Radiologia, Amadora, Lisboa, Portugal.

**Keywords:** Cateterismo Venoso Central, Diálise Renal, Veia Cava Superior, Cateteres, Cateterismo Venoso Central, Diálise Renal, Veia Cava Superior, Cateteres

## Abstract

Hemodialysis central venous catheter (CVC) insertion can be complicated in patients with anomalous vessel anatomy. In such cases detailed knowledge of thoracic vessel anatomy is necessary to identify the exact location of the catheter. Central venous placement under ultrasound control has signiﬁcantly reduced the complications associated with blind puncture and allows an appropriate puncture of the desired vessel, but the CVC can still get misplaced if it follows an anomalous route. Herein, we report a case of dialysis catheter placed into a left sided superior vena cava, only diagnosed after CT scan study.

## Resumo

A inserção do cateter venoso central (CVC) para hemodiálise pode ser complicada em pacientes com anatomia anômala dos vasos. Nesses casos, o conhecimento detalhado da anatomia do vaso torácico é necessário para identificar a localização exata do cateter. A colocação venosa central sob controle de ultrassom tem reduzido significativamente as complicações associadas à punção às cegas e permite uma punção apropriada do vaso desejado, mas o CVC ainda pode ficar mal posicionado se seguir uma rota anômala. Aqui, relatamos um caso de cateter de diálise colocado em uma veia cava superior esquerda, apenas diagnosticado após estudo de tomografia computadorizada.

## Introduction

Although arteriovenous fistulae remain the vascular access of choice for patients on chronic hemodialysis, up to 80% of patients initiate hemodialysis (HD) by central venous catheter (CVC) and, in some countries, up to 45% of patients prevalent on dialysis use this vascular access^1^.

CVC insertion is associated with risk, and complications may occur during the procedure, such as inadvertent arterial puncture, hemorrhage, hemopericardium, air embolism or hemo- and pneumothorax, immediately after its placement, such as catheter tip malposition, or late, such as central vein stenosis, thrombosis or infections^2^^,^ 3. According to previous publications, primary catheter malposition has an incidence between 1.8% and 3.7% and left side procedures have a significantly higher risk of catheter tip malposition, which may be as high as 30%^2^^,^ 4. Although ultrasound guidance allows a proper vessel puncture, it cannot help in following or directing the catheter trajectory.

Despite the skill level of the operator and the use of ultrasound guidance, CVC placement can result in CVC malposition and this can be associated with significant morbidity and mortality.

## Case report

An 80-year-old male patient on chronic hemodialysis was admitted after arteriovenous fistula thrombosis with no possibility of surgical recovery. He had no history of previous CVC. After ultrasound evaluation of vascular cervical structures and confirmation of right internal jugular vein (IJV) position and patency, it was decided to place a tunneled catheter.

During the procedure, the vein puncture was easy and the guidewire was passed without difficulty. A peel-away introducer was passed over the guidewire, the wire was removed, and the dialysis catheter was tunneled from a suitable site in the patient's chest. There was a slight difficulty in passing the catheter through the introducer, but it was possible to insert it with gentle movements and the introducer was peeled off. The lumens were flushed with some resistance in the venous side, and the catheter needed to be rotated and slightly repositioned to obtain adequate blood flow.

**Figure 1:**
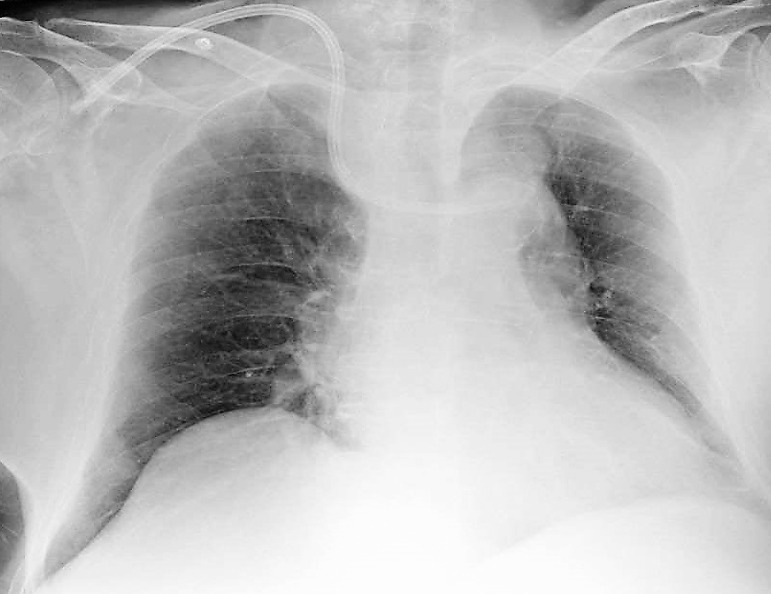
Anterior-posterior chest radiograph. The tip of the catheter is in a left sided position. There is widening of the mediastinum with a regular pale opacity above the aortic knob.

The procedure seemed uneventful, and a routine X-ray was performed showing the catheter tip in a left side vessel at the 3^rd^ rib level (Figure 1). A wide mediastinum and a regular, pale, opaque image were also noticed above the aortic knob.

**Figure 2:**
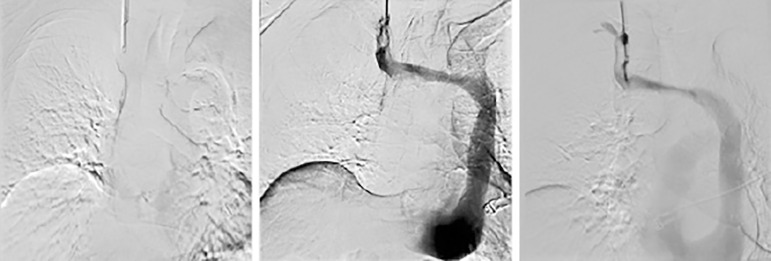
Fluoroscopy. The tunneled catheter was partially removed until the tip was positioned in the right IJV and contrast was injected revealing absence of luminal permeability of right superior vena cava.

An attempt to reposition the catheter was performed under fluoroscopy, unsuccessfully (Figure 2). The tunneled catheter was partially removed until the tip was positioned in the right IJV and contrast was injected revealing absence of the right superior vena cava. Then, the tunneled catheter was removed. These findings prompted further investigation.

**Figure 3:**
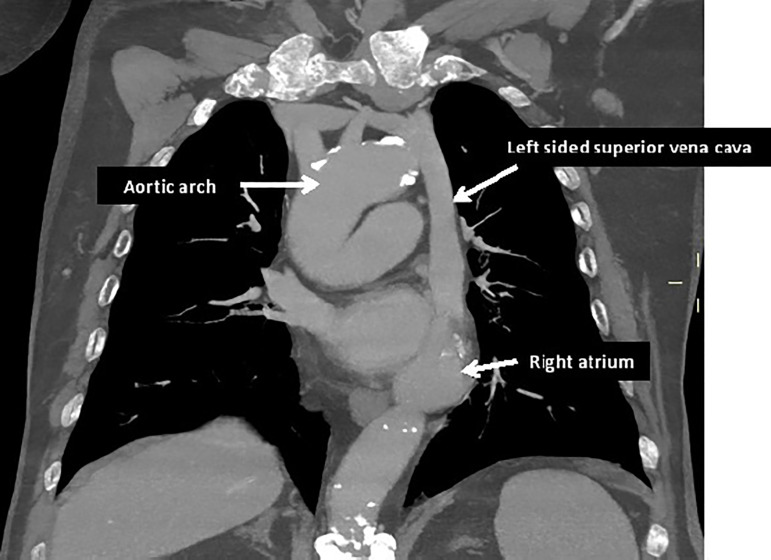
CT scan: Coronal MPR MIP image. Right IJV continues to the left where it receives the left IJV to form a left sided superior vena cava.

A cervical and thoracic CT scan was obtained showing the right IJV continuing to the left and receiving the left IJV to form a left sided superior vena cava. Therefore, a congenital anomaly with persistence of superior left vena cava with concomitant absence of superior right vena cava was diagnosed (Figure 3).

A new tunneled hemodialysis catheter was placed in the right femoral vein position.

## Discussion

In patients with superior vena cava obstruction, the tip of the catheter is often seen in collateral mediastinal venous pathways, rather than in the superior vena cava. The routine use of a ﬂuoroscope is recommended for placement of complicated dialysis catheters to avoid serious complications.

In this particular case, the vein puncture was easy and the guidewire was passed without difficulty but the catheter insertion was made under some resistance. After guidewire insertion in the vessel, it is crucial to enlarge the cutaneous puncture site with scalpel in order to facilitate the insertion of dilators and to prevent kinking of the catheter after passing it through the sheath. A deficient dissection of the subcutaneous space between the skin surface and the vessel wall may be responsible for the resistance encountered when inserting the HD catheter through the introducer.

However, difficulty encountered during CVC insertion can also be due to stenosis or thrombosis. In addition, variations in venous anatomy can lead to resistance at some point during the passing of the catheter into tributary veins that usually offer low-resistance routes for catheter tip entrance.

Venous anatomy varies greatly, and two types of variants are highlighted: congenital and acquired. In patients with CVCs, congenital variants are usually discovered incidentally on imaging after CVC placement^5^ .

The procedure of the present case was correctly performed. However, as radiologic studies revealed, the patient had an anatomic venous variant with total absence of the right superior vena cava (SVC) and persistence of the left SVC.

The persistent left SVC is a common congenital variation with no clinical significance, which is seen in 0.3% of healthy patients and 4.3% of patients with congenital heart disease^6^^,^ 7. The most common subtype of persistent left SVC results in the presence of both left and right SVCs^8^^,^ 9. More rarely, abnormalities of the embryological development lead to an absent right SVC with persistent left SVC, as previously described in both pediatric and adult patients^10^^-^ 16. In most cases, persistent left SVC drains into the right atrium via the coronary sinus with of no hemodynamic consequence^17^.

The presence of associated anomalies such as atrial septal defect, bicuspid aortic valve, coarctation of aorta, coronary sinus ostial atresia, or cor triatriatum is more common with concomitant absence of right SVC^18^.

The higher incidence of malposition in the left thoracic venous system than in the right side has been already documented, which suggests that the right side should be considered the first choice for CVC insertion unless contraindicated.

Placement of HD catheters in right IJV is a common procedure for most nephrologists. To prevent complications related to vascular anatomic variants, a careful history about previous catheters placement should be obtained. Additionally, ultrasound guidance should be used in all procedures. However, even after easy insertion of the guidewire some precautions should be taken, since there is risk of perforation due to direct injury from the dilator. The dilators should only be inserted deep enough to allow the sheath to be placed.

Although plain chest radiography is the standard imaging modality for confirming catheter tip location, signs and symptoms of CVC malposition even in presence of normal or inconclusive conventional radiography findings should prompt the use of additional diagnostic methods to confirm or rule out the diagnosis. Verification of guidewire direction under fluoroscopic guidance is also recommended by some authors for tunneled dialysis catheters^19^. Recently, Jheengut and Fan described the utility of intracavitary electrocardiogram to identify the persistency of left SVC^20^. With very few exceptions, the recommendation in cases of intravascular CVC malposition is to remove and relocate the catheter.

## References

[1] Allon M (2019). Quantification of complications of tunneled hemodialysis catheters. Am J Kidney Dis.

[2] Weber E, Liberek T, Wolyniec W, Rutkowski B (2015). Catheter tip malposition after percutaneous placement of tunneled hemodialysis catheters. Hemodial Int.

[3] Premuzic V, Smiljanic R, Perkov D, Gavranic BB, Tomasevic B, Jelakovic B (2016). Complications of permanent hemodialysis catheter placement; need for better pre-implantation algorithm . Ther Apher. Dial.

[4] Muhm M, Sunder-Plassmann G, Apsner R (1997). Malposition of central venous catheters Incidence, management and preventive practices. Wien Klin Wochenschr.

[5] Demos TC, Posniak HV, Pierce KL, Olson MC, Muscato M (2004). Venous anomalies of the thorax. Am J Roentgenol.

[6] Povoski SP, Khabiri H (2011). Persistent left superior vena cava review of the literature, clinical implications, and relevance of alterations in thoracic central venous anatomy as pertaining to the general principles of central venous access device placement and venography in cancer. World J Surg Oncol.

[7] Pahwa R, Kumar A (2003). Persistent left superior vena cava ah intensivist's experience and review of the literature. South Med J.

[8] Liberek T, Swiader W, Koprowski A, Bascik B, Debska-Slizien A (2020). Tunnelled haemodialysis catheter insertion into the persistent left superior vena cava. J Vasc Access.

[9] Zhou Q, Murthy S, Pattison A, Werder G (2016). Central venous access through a persistent left superior vena cava: a case series. J Vasc Access.

[10] Ricciardi B, Ricciardi CA, Lacquaniti A, Carella G, Puzzolo D, Pisani A (2017). Persistent left superior vena cava and partially left inferior vena cava: a case report of a dangerous central venous catheterization. J Vasc Access.

[11] Bulsara SS, Paliwal V, Prasad G, Bharath M, Sahu T, Sheorain V (2018). Vascular access in a rare case of 'isolated-persistent left superior vena cava' J Vasc. Access.

[12] Mandolfo S, Maggio M, Bucci R, Borlandelli S, Ronga C, Farina M (2017). Contrast echocardiographic and persistent left superior vena cava. J Vasc Access.

[13] Caruselli M, Piattellini G, Camilletti G, Giretti R, Pagni R (2009). Persistent left superior vena cava in pediatric patients. J Vasc Access.

[14] Parreira LF, Lucas CC, Gil CC, Barata JD (2009). Catheterization of a persistent left superior vena cava. J Vasc Access.

[15] Granata A, Andrulli S, Fiorini F, Logias F, Figuera M, Mignani R (2009). Persistent left superior vena cava: What the interventional nephrologist needs to know. J Vasc Access.

[16] Dionisio P, Borsetti C, Valenti M, Caramello E, Bergia R, Berto IM (2003). Knowledge of the anomalies of the big central veins reduces the morbidity during the cannulation for hemodialysis: description of a case of persistent left superior vena cava and revision of literature. J Vasc Access.

[17] Goyal SK, Punnam SR, Verma G, Ruberg FL (2008). Cardiovascular ultrasound persistent left superior vena cava: a case report and review of literature. Cardiovasc Ultrasound.

[18] Sarodia BD, Stoller JK (2000). Persistent left superior vena cava case report and literature review. Respir Care.

[19] Rossi UG, Rigamonti P, Torcia P, Mauri G, Brunini F, Rossi M (2015). Congenital anomalies of superior vena cava and their implications in central venous catheterization. J Vasc Access.

[20] Jheengut Y, Fan B (2020). Intraoperative identification of persistent left superior vena cava with intracavitary electrocardiogram during venous port insertion: a report of eight cases. J Vasc Access.

